# Popliteal Aneurism; Treatment by Flexion; Cure

**Published:** 1880-11

**Authors:** 


POPLITEAL ANEURISM; TREATMENT BY FLEXION; CURE. (Under the care of Mr. NEWNHAM.)

Leorge L., aged forty-four, a goods-guard, was admitted with popliteal aneurism. His previous history was excellent: he had never had syphilis, and had always been abstemious. Several months before he noticed the aneurism he wrenched his knee, and had to lay up for a short time. He could remember no other injury to the leg, nor could he recollect having made a forcible or sudden muscular effort to lift weights.

The history of the aneurism was as follows: Three weeks before admission (since January 10), the patient noticed a pulsation in the left popliteal space. His attention was directed to the seat of disease by pain in the calf when walking, the pain passing off if he stood still even for a moment. He thought that the swelling when he first noticed it was nearly as large as on admission.

On January 31 he was taken in under the care of Mr. Newnham. In the left popliteal space there was a tumor, about the size of a hen's egg. It had an extensile pulsation which could be arrested by pressure on the femoral artery. The area of pulsation extended over the popliteal space. There was a distinct bruit, and a thrill was communicated to the hand. On examining the other arteries of the body, the left radial and temporal arteries were found feebler than the corresponding arteries on the opposite side, but no intrathoracic tumor could be detected. The superficial arteries had not an atheromatous feel, the heart was healthy, and no visceral disease could be discovered. The patient had a cataract of the right eye, which did not result from injury. He was a corpulent man, and somewhat aged in appearance, but his health, he said, was exceedingly good.

The patient was put on meat diet, and given five grains of the iodide of potassium and fifteen of the bicarbonate of potash. He was kept in bed and had an occasional purge. On February 4, he was directed to keep his thigh flexed on the abdomen, and the leg on the thigh; the iodide was increased to ten grains. On the 6th, he was put on a modified Bellingham's diet. Breakfast: bread and butter, two ounces; milk, two ounces. Dinner: meat, two ounces; bread, two ounces; beer, two ounces. Supper: bread and butter, two ounces; milk, two ounces. On the 8th, the extensile pulsation had ceased in the tumor, although the pulsation in the popliteal and posterior tibial arteries could be felt. On the 9th, the angle of flexion was increased; daily allowance of fluid, fourteen ounces. On the 11th, flexion was discontinued; patient put on meat diet, with one pint of fluid. The aneurismal sac was felt to the inner side of the popliteal artery; it was a little larger than a walnut, and felt solid to the touch. The patient was left in bed for ten days after pulsation had ceased, and was discharged on the 9th of March, when the tumor could be scarcely felt.

